# Supplementary material for: The Impact of Genetics on Gut Microbiota of Growing and Fattening Pigs under Moderate N Restriction
Source: Animals (Basel). 2021 Sep 29;11(10):2846. doi: 10.3390/ani11102846 (PMC8532768; doi:10.3390/ani11102846)
Supplement: Supplementary file 1 [file animals-11-02846-s001.zip › animals-1385261-supplementary.pdf]

Supplementary Material: Role of Genetics in the Composition of Pig Gut Microbiota Under Nitrogen Restriction in Growing and Fattening Phases

Laura Sarri, Sandra Costa-Roura, Joaquim Balcells, Ahmad Reza Seradj and Gabriel de la Fuente *

Table S1. Microbial alpha diversity indices (based on OTUs), and Firmicutes/Bacteroidetes ratio (Ratio F/B) in ileum, cecum, and distal colon segments.

Item [1]	Growing phase		Fattening phase		SEM	P-value	
	SP	LP	SP	LP		Diet	PP
n	12	8	8	8			
Ileum [2]							
Shannon index	1.60	1.57	1.86	1.79	0.160	0.640	0.186
Simpson index	0.66	0.65	0.73	0.74	0.049	0.855	0.102
Richness	26.98	28.88	37.13	38.25	3.854	0.845	0.020
Evenness	0.50	0.47	0.52	0.49	0.040	0.381	0.785
Cecum							
Shannon index	3.18	3.35	3.56	3.42	0.177	0.943	0.189
Simpson index	0.92	0.92	0.92	0.93	0.018	0.776	0.618
Richness	95.88	99.25	127.25	113.75	8.973	0.572	0.013
Evenness	0.70	0.73	0.74	0.74	0.034	0.629	0.457
Ratio F/B	48.16	23.81	7.76	30.30	18.409	0.904	0.309
Distal colon							
Shannon index	3.65	3.72	3.66	3.79	0.128	0.394	0.650
Simpson index	0.94	0.95	0.94	0.95	0.009	0.546	0.742
Richness	134.14	129.50	151.97	147.88	9.595	0.634	0.045
Evenness	0.75	0.76	0.76	0.76	0.017	0.597	0.820
Ratio F/B	27.09	12.55	10.06	9.08	8.710	0.254	0.112

[1] Obtained in pigs differing in their protein intake (Diet): standard protein (SP) vs low protein (LP) and in their production phase (PP): growing (28.5 kg) vs fattening (88.1 kg). Standard error of the mean (SEM) and significance of Diet and PP effects are shown. Mean values within a row followed by different letters differ significantly at $P = 0.05$. No significant Diet by PP interaction was found and their values were not included in the table.

[2] Ratio F/B could not be calculated in ileum samples due to the low abundance of Bacteroidetes phylum in that intestinal tract (0.28% of analyzed sequences).

Table S2. Microbial alpha diversity indices (based on OTUs).

Item[1]	Growing phase			Fattening phase			SEM	P-value	
	Ileum	Cecum	Distal colon	Ileum	Cecum	Distal colon		Segment	PP
n	20	20	20	16	16	16			
Shannon index	1.58c	3.23b	3.65ab	1.82c	3.49ab	3.72a	0.116	<0.001	0.032
Simpson index	0.65c	0.91a	0.95a	0.74b	0.91a	0.95a	0.023	<0.001	0.084
Richness	28.45c	95.75b	130.65a	37.69c	120.50a	142.38a	6.119	<0.001	0.002
Evenness	0.49b	0.71a	0.75a	0.52b	0.74a	0.76a	0.023	<0.001	0.124

[1] Samples were obtained from three intestinal segments (ileum, cecum and distal colon) in pigs differing in their production phase (PP): growing (28.5 kg) vs fattening (88.1 kg). Standard error of the mean (SEM) and significance of intestinal Segment and PP effects are shown. Mean values within a row followed by different letters differ significantly at $P = 0.05$. No significant Segment by PP interaction was found and their values were not included in the table.

Table S3. Network metrics in the ileum, cecum and distal colon of Duroc and F2 pigs, fed diets of standard (SP) and low (LP) crude protein contents in the growing and fattening phases.

Item	Growing phase				Fattening phase			
	Duroc	F2	SP	LP	Duroc	F2	SP	LP
Ileum								
Nodes	14	16	12	14	9	9	8	11
Edges	19	21	14	19	11	9	5	9
Node degree	2.71	2.63	2.33	2.71	2.44	2.00	1.25	1.64
Betweenness	0.79	0.38	0.67	0.64	0.22	4.00	0.13	0.64
Cecum								
Nodes	43	56	43	53	50	53	52	52
Edges	117	157	81	144	106	270	185	180
Node degree	5.44	5.61	3.77	5.43	4.24	10.19	7.12	6.92
Betweenness	4.88	6.91	1.65	3.79	6.16	11.19	8.19	6.62
Colon								
Nodes	66	70	66	71	64	71	67	72
Edges	146	312	176	204	137	417	177	270
Node degree	4.42	8.91	5.33	5.75	4.35	11.75	5.28	7.50
Betweenness	4.42	15.69	5.38	9.59	4.90	20.89	4.82	16.15

Network interactions analysis were performed through Sparse Correlations for Compositional data (SparCC) technique (R Core Team, 2020; SpiecEasi package) over those genera present at least at 50% of the individuals. Their complexity was described in terms of number of nodes (genera) and edges (significant positive or negative correlations), node degree (number of connections that any node establishes with other nodes) and betweenness centrality (measure of centrality in a graph based on shortest paths).

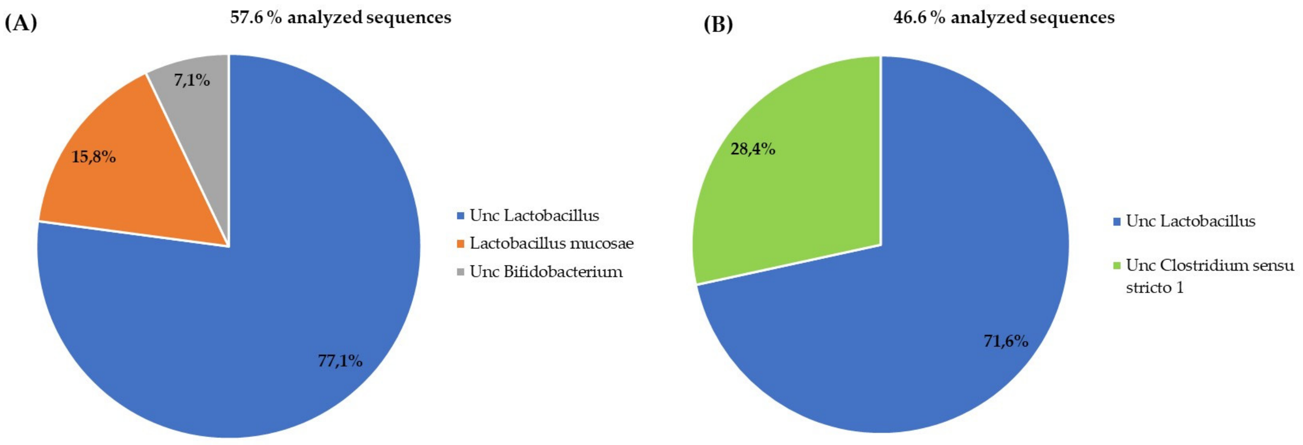

Figure S1. Microbial core community composition (OTUs) in ileum of Duroc and F2 (Pietrain × F1: Duroc × Landrace) pigs in either growing (A, 28.5 kg) or fattening (B, 88.1 kg) production phases. Unc: unclassified.

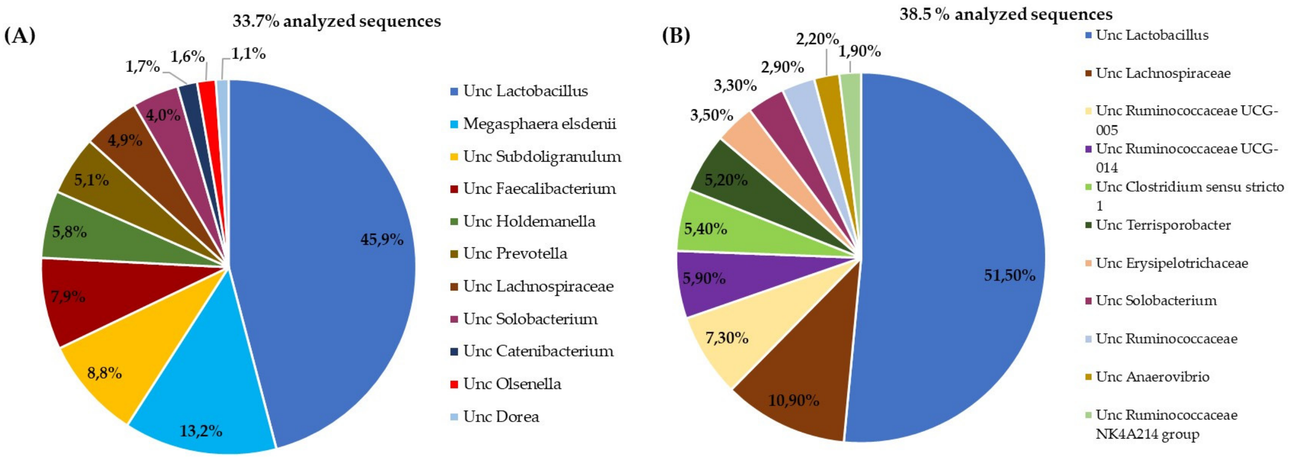

Figure S2. Microbial core community composition (OTUs) in cecum of Duroc and F2 (Pietrain × F1: Duroc × Landrace) pigs in either growing (A, 28.5 kg) or fattening (B, 88.1 kg) production phases. Unc: unclassified.

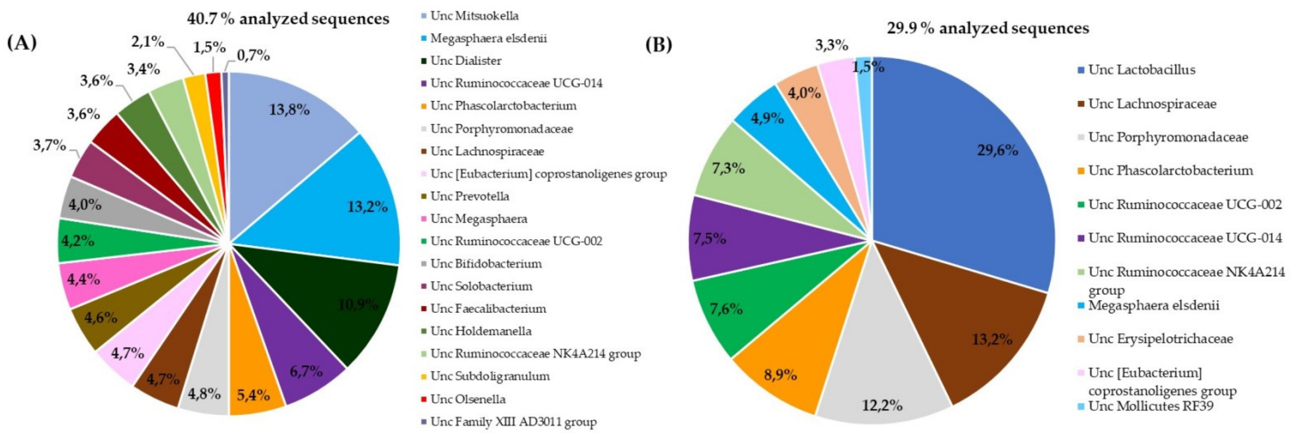

Figure S3. Microbial core community composition (OTUs) in distal colon of Duroc and F2 (Pietrain × F1: Duroc × Landrace) pigs in either growing (A, 28.5 kg) or fattening (B, 88.1 kg) production phases. Unc: unclassified.

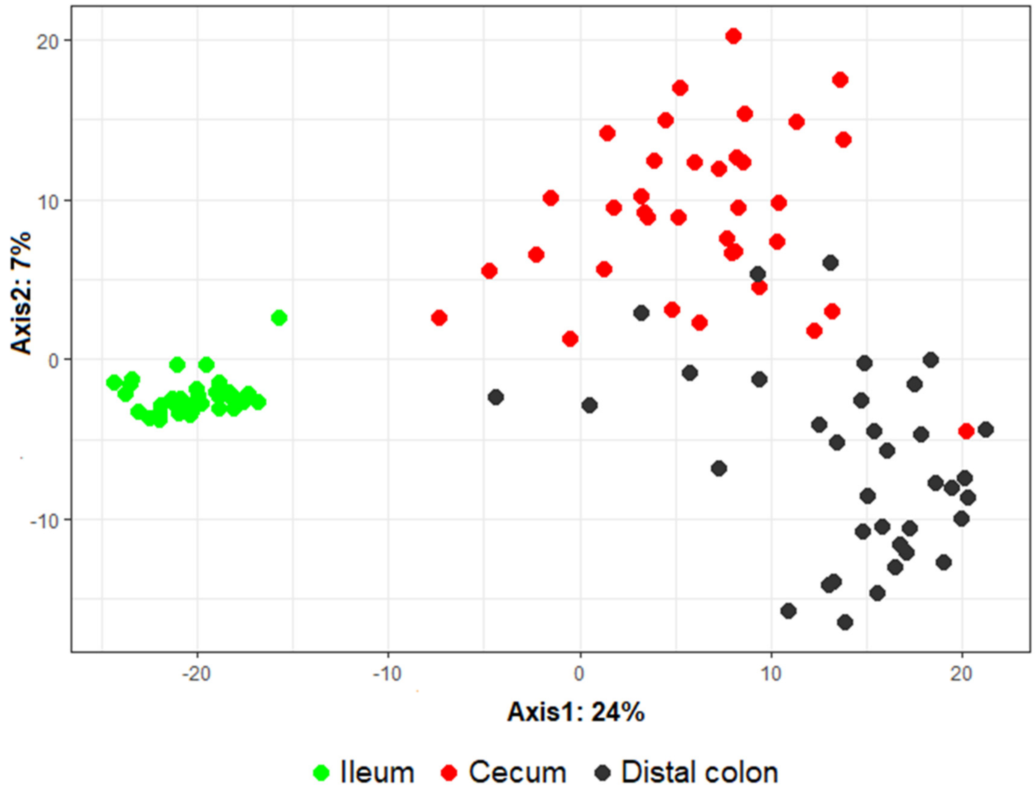

Figure S4. Graphical representation of partial least squares-discriminant analysis (PLS–DA) on microbial OTUs in ileum, cecum and distal colon. Each point represents a different sample and a greater distance between two points infers a higher dissimilarity between them. Samples clearly clustered by intestinal segment, indicating that ileum, cecum and distal colon harbored different microbial communities (PERMANOVA $P < 0.001$).

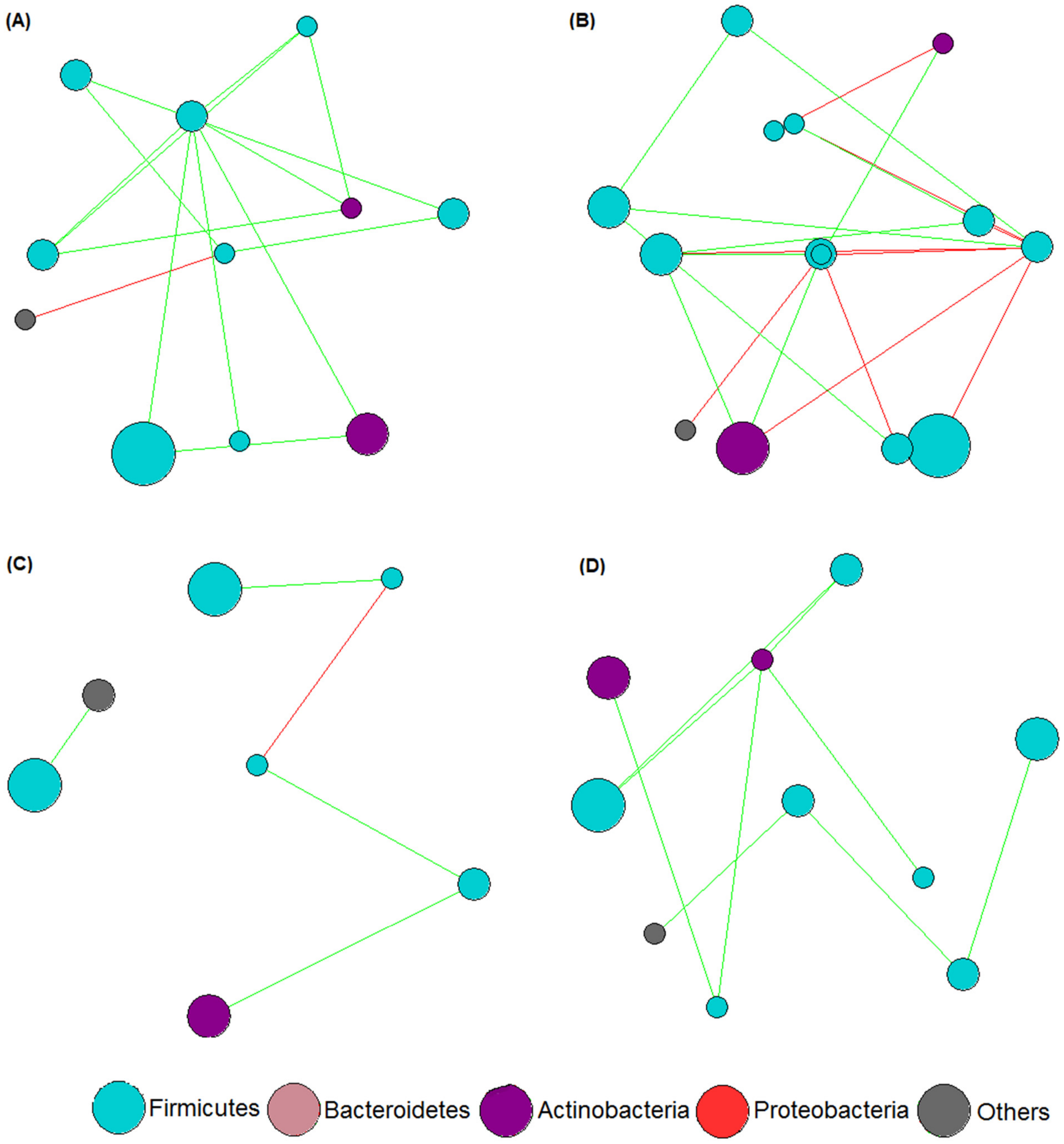

Figure S5. Microbial genera network in ileum. Obtained in pigs differing in their protein intake: (A–C) standard protein vs (B–D) low protein and production phase: (A–B) growing (28.5 kg) vs (C–D) fattening (88.1 kg). Green and red edges indicate positive and negative correlations, respectively. Node size is proportional to genera abundance and node color indicates phyla affiliation. Minor phyla are represented as “Others”.

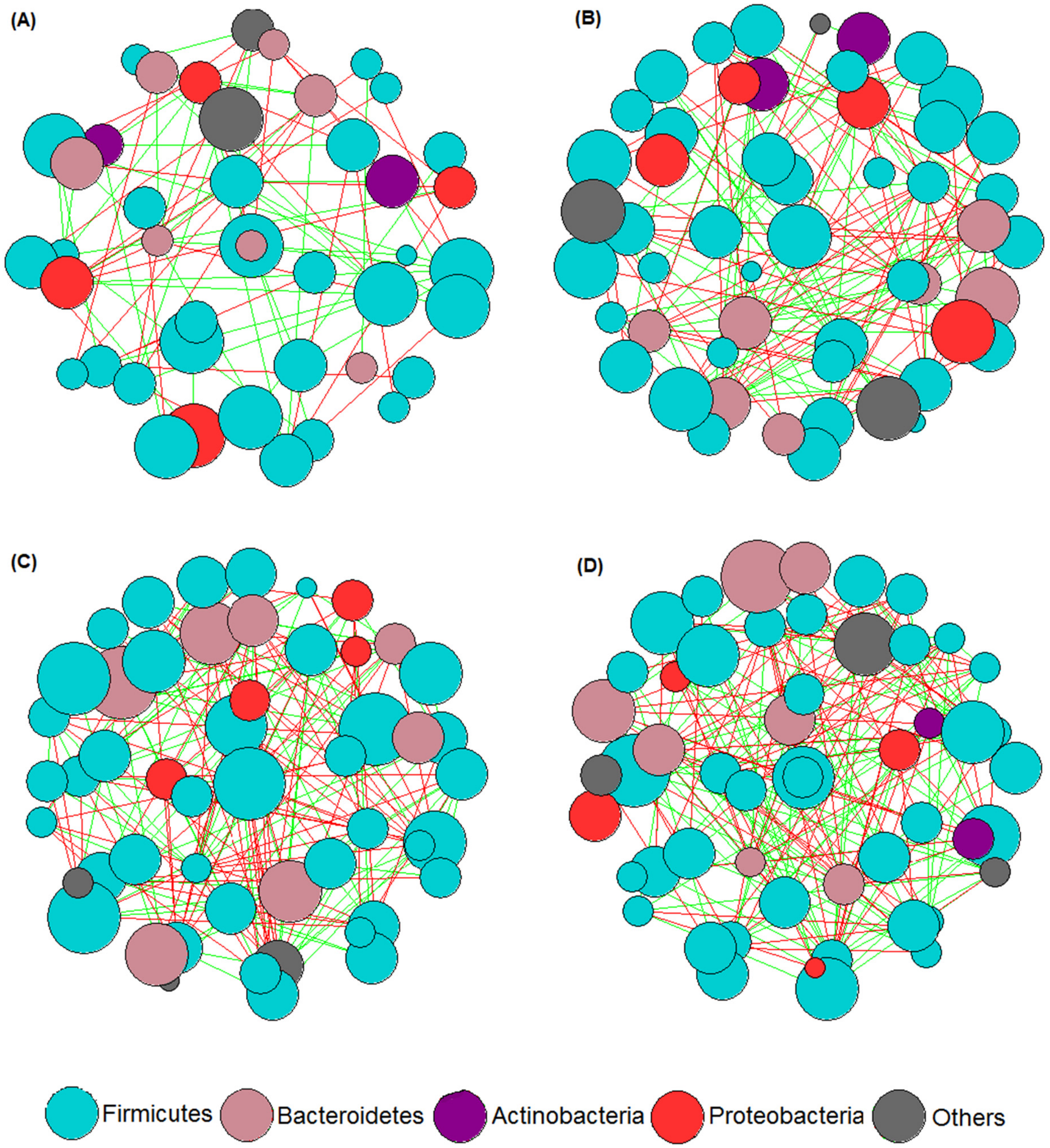

Figure S6. Microbial genera network in cecum. Obtained in pigs differing in their protein intake: (A–C) standard protein vs (B–D) low protein and production phase: (A–B) growing (28.5 kg) vs (C–D) fattening (88.1 kg). Green and red edges indicate positive and negative correlations, respectively. Node size is proportional to genera abundance and node color indicates phyla affiliation. Minor phyla are represented as "Others".

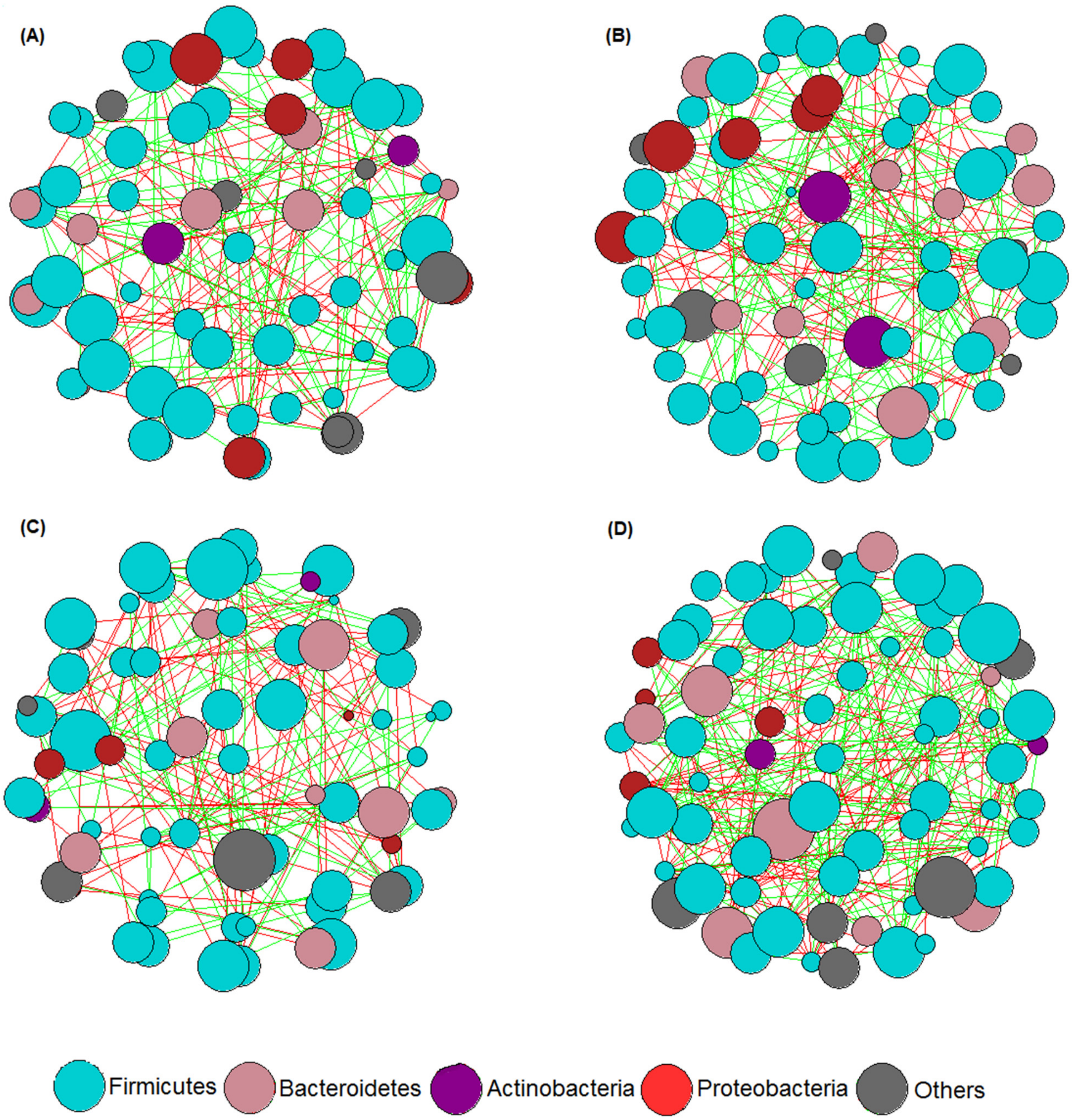

Figure S7. Microbial genera network in distal colon. Obtained in pigs differing in their protein intake: (A–C) standard protein vs (B–D) low protein and production phase: (A–B) growing (28.5 kg) vs (C–D) fattening (88.1 kg). Green and red edges indicate positive and negative correlations, respectively. Node size is proportional to genera abundance and node color indicates phyla affiliation. Minor phyla are represented as "Others".

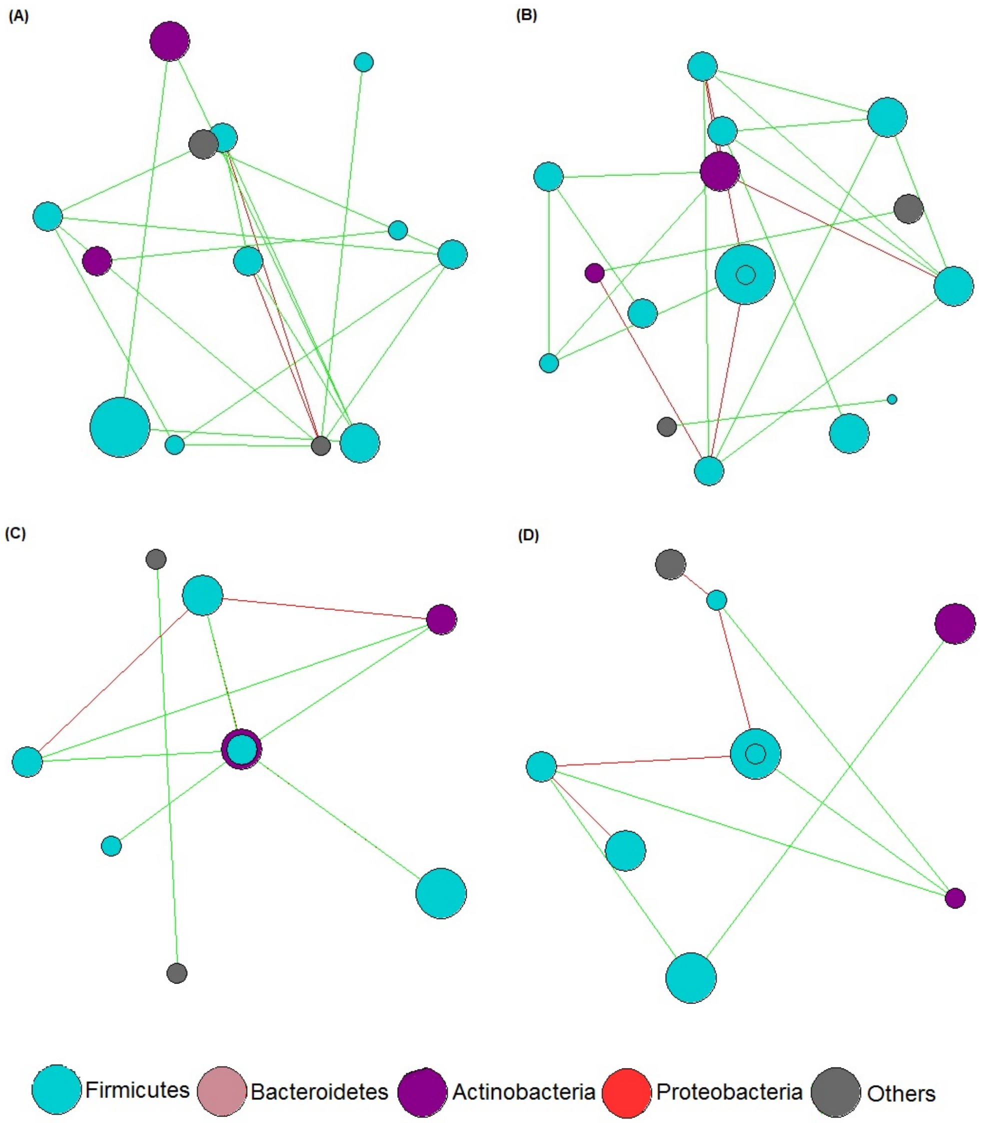

Figure S8. Microbial genera network in ileum. Obtained in pigs differing in their production type: (A–C) Duroc vs (B–D) F2 (Pietrain × F1: Duroc × Landrace) and production phase (A–B) growing (28.5 kg) vs (C–D) fattening (88.1 kg). Green and red edges indicate positive and negative correlations, respectively. Node size is proportional to genera abundance and node color indicates phyla affiliation. Minor phyla are represented as "Others".

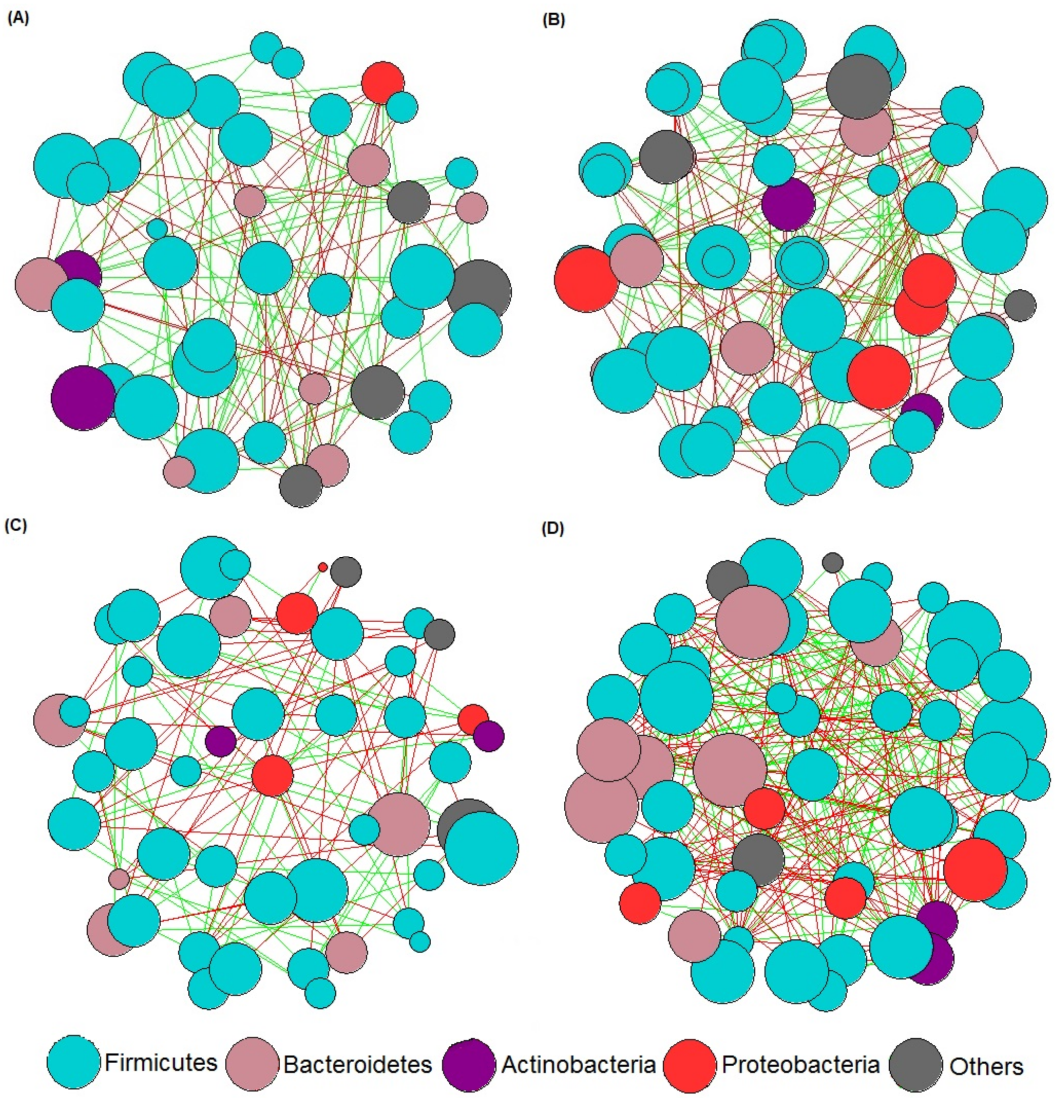

Figure S9. Microbial genera network in cecum. Obtained in pigs differing in their production type: (A–C) Duroc vs (B–D) F2 (Pietrain × F1: Duroc × Landrace) and production phase (A–B) growing (28.5 kg) vs (C–D) fattening (88.1 kg). Green and red edges indicate positive and negative correlations, respectively. Node size is proportional to genera abundance and node color indicates phyla affiliation. Minor phyla are represented as "Others".

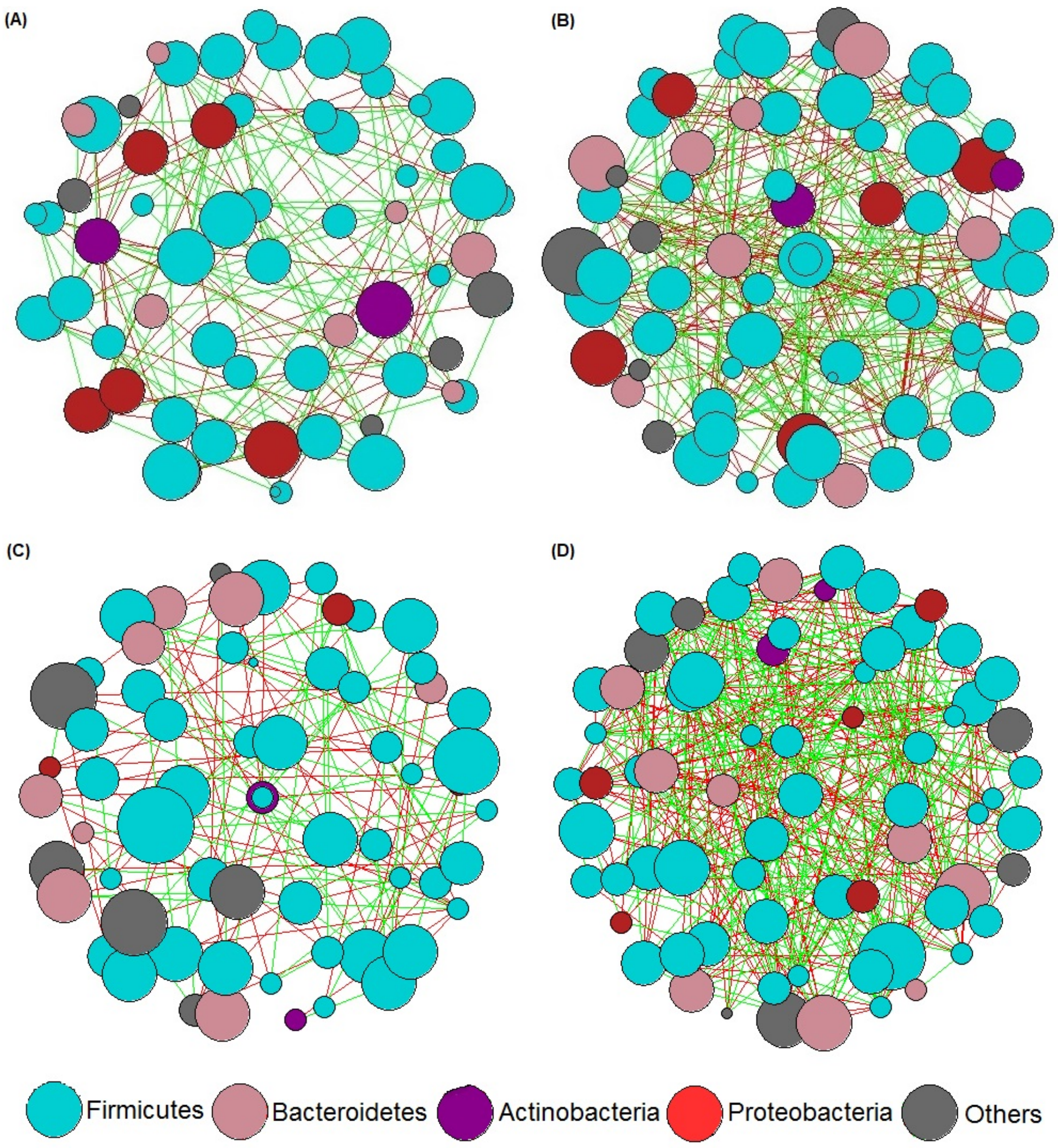

Figure S10. Microbial genera network in distal colon. Obtained in pigs differing in their production type: (A–C) Duroc vs (B–D) F2 (Pietrain × F1: Duroc × Landrace) and production phase (A–B) growing (28.5 kg) vs (C–D) fattening (88.1 kg). Green and red edges indicate positive and negative correlations, respectively. Node size is proportional to genera abundance and node color indicates phyla affiliation. Minor phyla are represented as "Others".
